# Self-Assembled Nanoparticles of Glycyrrhetic Acid-Modified Pullulan as a Novel Carrier of Curcumin

**DOI:** 10.3390/molecules190913305

**Published:** 2014-08-28

**Authors:** Roufen Yuan, Fuchun Zheng, Shuping Zhong, Xiaojun Tao, Yanmei Zhang, Fenfei Gao, Fen Yao, Jiaxiong Chen, Yicun Chen, Ganggang Shi

**Affiliations:** 1 Department of Pharmacology, Shantou University Medical College, Shantou 515041, China; 2 Department of Pharmacy, First Affiliated Hospital, Shantou University Medical College, Shantou 515041, China; 3 Department of Biochemistry and Molecular Biology, Keck School of Medicine, University of Southern California, Los Angeles, CA 90033, USA; 4 Medical College, Hunan Normal University, Changsha 410013, China; 5 Chinese Academy of Sciences, Shantou Marine Plants Experiment Station, Shantou 515041, China; 6 Traditional Chinese Medicine Laboratory, Shantou University Medical College, Shantou 515041, China; 7 Department of Cardiovascular Diseases, First Affiliated Hospital, Shantou University Medical College, Shantou 515041, China

**Keywords:** pullulan, glycyrrhetic acid, self-aggregated nanoparticle, curcumin, drug delivery

## Abstract

Glycyrrhetic acid (GA)-modified pullulan nanoparticles (GAP NPs) were synthesized as a novel carrier of curcumin (CUR) with a degree of substitution (DS) of GA moieties within the range of 1.2–6.2 groups per hundred glucose units. In the present study, we investigated the physicochemical characteristics, release behavior, *in vitro* cytotoxicity and cellular uptake of the particles. Self-assembled GAP NPs with spherical shapes could readily improve the water solubility and stability of CUR. The CUR release was sustained and pH-dependent. The cellular uptake of CUR-GAP NPs was confirmed by green fluorescence in the cells. An MTT study showed CUR-GAP NPs with higher cytotoxicity in HepG2 cells than free CUR, but GAP NPs had no significant cytotoxicity. GAP is thus an excellent carrier for the solubilization, stabilization, and controlled delivery of CUR.

## 1. Introduction

Curcumin (CUR), a low-molecular-weight and hydrophobic polyphenol with fluorescence, obtained from the turmeric *Curcuma* rhizome, has low intrinsic toxicity but a wide range of pharamacological activities, including antioxidant, anti-inflammatory, antimicrobial, antiamyloid, and antitumor properties [1,2]. CUR has great promise as a chemopreventive and therapeutic agent in liver cancer—It significantly attenuated tumor growth in female BALB/c athymic mice injected with Bel7402, SGC7901 and HL60 cells [3] and showed antiangiogenic activity in male BALB/c nude mice implanted with human HepG2 cells [4], possibly because of its potent antioxidant and anti-inflammatory properties and its ability to modulate a multitude of signaling mechanisms [5].

However, its extremely low aqueous solubility, rapid systemic elimination, inadequate tissue absorption and degradation at alkaline pH severely curtail the clinical application of CUR [6,7,8]. As a solution attempts have been made to increase the aqueous solubility and bio-availability by encapsulation of CUR in polymeric micelles, liposomes, polymeric nanoparticles (NPs), lipid-based NPs, and hydrogels [9,10,11,12,13,14].

Nanoparticulate drug delivery systems are used to alter biodistribution, target desired cells, and control the release of chemotherapeutic drugs, an important approach with great potential for overcoming problems associated with the systemic toxicity of chemotherapy [15]. Pullulan-based NPs have been extensively used for nanoparticulate drug delivery because of their outstanding biocompatibility, high water-solubility, non-toxicity, and multiple hydroxyl groups that can easily be chemically modified; in addition, they lack immunogenicity, so are useful as a plasma expanders [16,17] and have inherent affinity for the liver [18,19,20]. Hydrophobized pullulan has been often used as a drug carrier; examples are cholesterol-bearing pullulan [21,22,23,24], poly(dl-lactide-co-glycolide)-grafted pullulan [25], and pullulan acetate [26]. 

Glycyrrhetinic acid (GA), the main bioactive compound in traditional Chinese medicine liquorice, possesses a wide range of pharmacological properties such as anti-inflammatory, antiviral, antimicrobial, antioxidative, and anticancer activities and immunomodulatory, hepatoprotective and cardioprotective effects [27,28,29]. Furthermore, GA is used as a liver-targeting ligand because some highly specific GA binding sites are located on the surface of liver parenchyma cells [30].

In this study, we synthesized a novel carrier based on GA-modified pullulan (GAP) with different degrees of substitution (DS) confirmed by ^1^H nuclear magnetic resonance (^1^H-NMR) and Fourier transform infrared spectroscopy (FT-IR). The self-assembled NPs were prepared by the dialysis method and evaluated by transmission electron microscopy (TEM) and dynamic light scattering (DLS). In addition, we evaluated the use of GAP NPs as a drug carrier of CUR and investigated release behavior, cellular uptake and cytotoxicity.

## 2. Results and Discussion

### 2.1. Synthesis and Characterization of GAP NPs

**Scheme 1:**
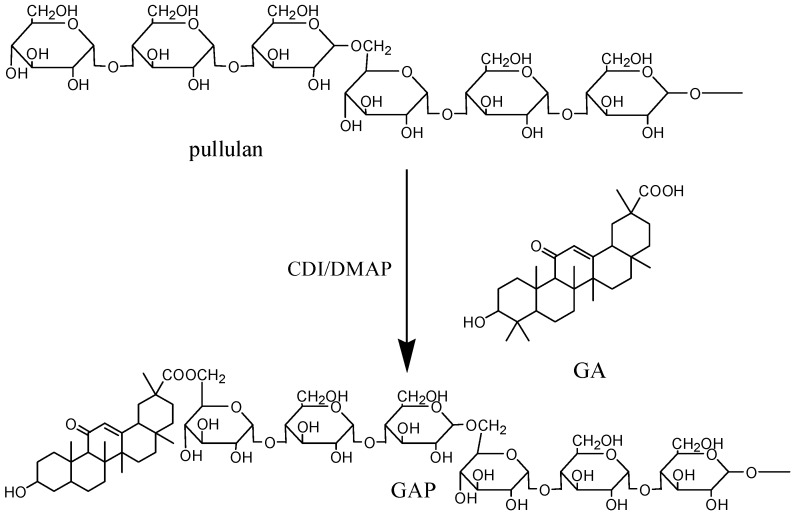
The synthesis of glycyrrhetic acid (GA)-pullulan (GAP).

The synthesis of GA-modified sulfated chitosan [31], O-carboxymethylchitosan [32], alginate [33] and hyaluronic acid [34] NPs *etc.* was reported recently. Chitosan, alginate and hyaluronic acid *etc.* polysaccharides and GA in these NPs need to be modified to increase water solubility and provide reactive groups. The syntheses of these NPs were complicated and tedious, which limits their industrial application and is not environmentally-friendly. However, pullulan can directly be modified by GA through a simple esterification step, because of its outstanding water-solubility and multiple hydroxyl groups. The synthesis of GAP is summarized in Scheme 1.

In the presence of DMAP and CDI, GA was attached to pullulan for a novel kind of polymeric amphiphile with different DS of GA moiety by controlling the molar ratio of GA to pullulan.

**Figure 1:**
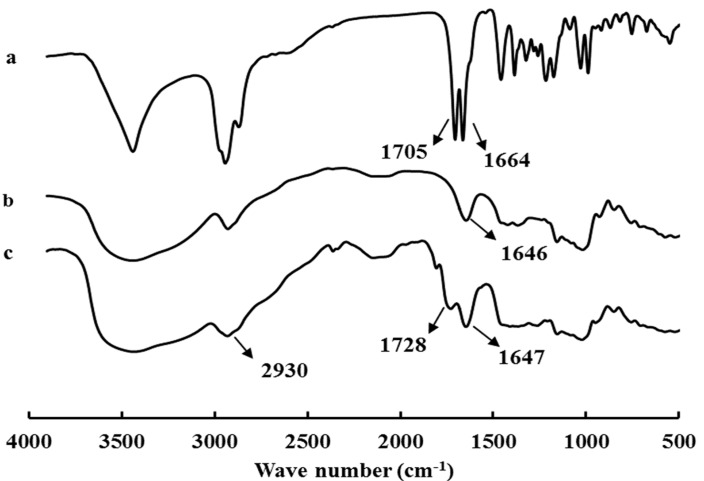
Fourier transform-infrared (FT-IR) spectra for (**a**) GA, (**b**) pullulan, and (**c**) GAP.

Peaks at 1728 cm^−1^ corresponding to carbonyl groups [35] appeared in the FT-IR spectra for GAP (Figure 1), so GA was covalently bound to pullulan; the enhanced stretching vibration band relative to -CH_3_, -CH_2_ groups of GA at about 2930 cm^−1^ confirmed this finding.

**Figure 2:**
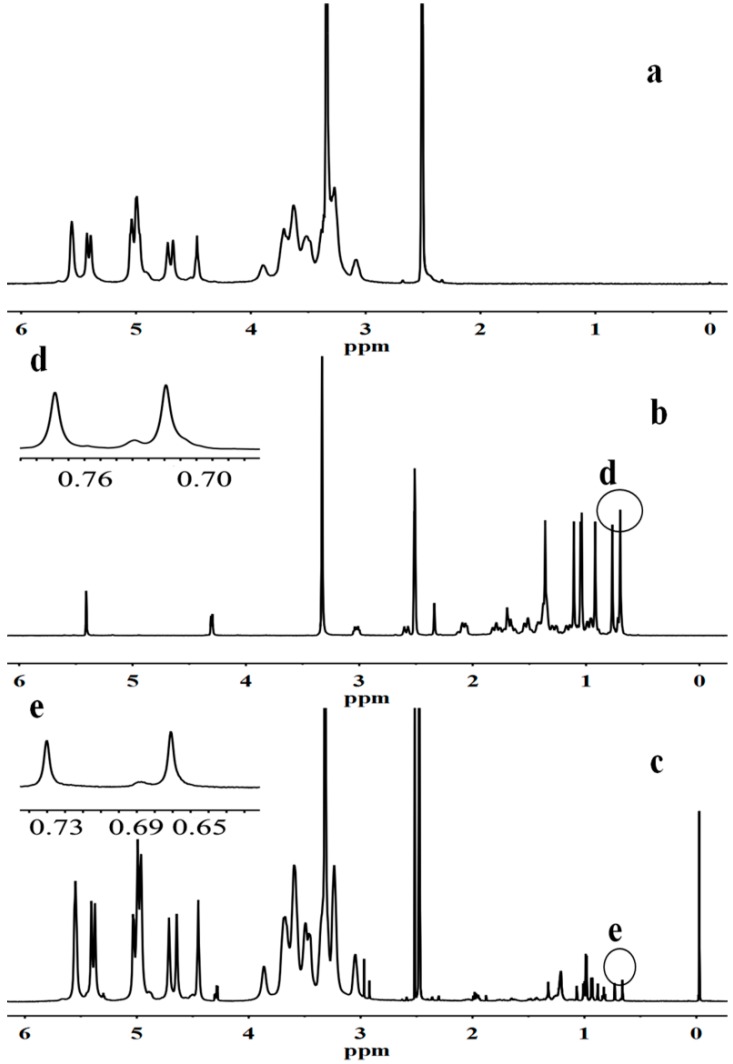
^1^H-NMR spectra for (**a**) pullulan, (**b**) GA, (**c**) GAP, (**d**) and (**e**) the angular methyl of C_23_ and C_24_.

The ^1^H-NMR spectra for GAP showed the characteristic peaks of pullulan and new peaks assigned to the CH_3_, CH_2_, and CH protons of the GA moiety at 0.63–2.0 ppm, which indicated the successful conjugation of GA to pullulan. Pullulan characteristic peak signals at 4.68 and 5.05 ppm correspond to the α-1,6 and α-1,4 glycosidic bonds protons of C_1_ [36], which could be identified as the glucose units in pullulan. The signals at 0.73 and 0.66 ppm corresponded to the protons of the C_23_ and C_24_ angular methyls in GA [37], whereby the two angular methyl peaks had almost the same shapes and positions of GA (Figure 2d,e). Therefore, the DS of GA residues per 100 glucose units for pullulan could be calculated by the ratio of angular methyl protons (0.73 and 0.66 ppm) of GA to sugar protons (C_1_ position of α-1, 6 and α-1, 4 glycosidic bonds) using the following equation:

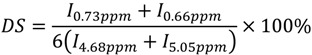
(1)

The results of the DS of GA moiety are in Table 1.

**Table 1:** The degree of substitution (DS) and diameter loading capacity (LC) and encapsulation efficiency (EE) of GAP NPs and CUR-GAP NPs. Data are mean ± SD, n = 3.

Samples	DS	Diameter of GAP NPs (nm)	Drug/Carrier (w/w %)	LC%	EE%	Diameter of CUR-GAP NPs (nm)
			5	4.97 ± 0.13	78.3 ± 3.05	97.1 ± 3.7
GAP1	6.2	63.7 ± 4.8	10	9.98 ± 0.28	72.7 ± 1.45	109.3 ± 8.6
			20	10.29 ± 0.21	68.9 ± 2.52	123.6 ± 9.5
GAP2	4.5	68.5 ± 5.3	10	6.75 ± 0.32	62.4 ± 3.10	132.7 ± 3.8
GAP3	1.2	82.1 ± 6.2	10	4.16 ± 0.17	48.3 ± 1.73	153.8 ± 6.2

### 2.2. Characterization and Drug Encapsulation

**Figure 3:**
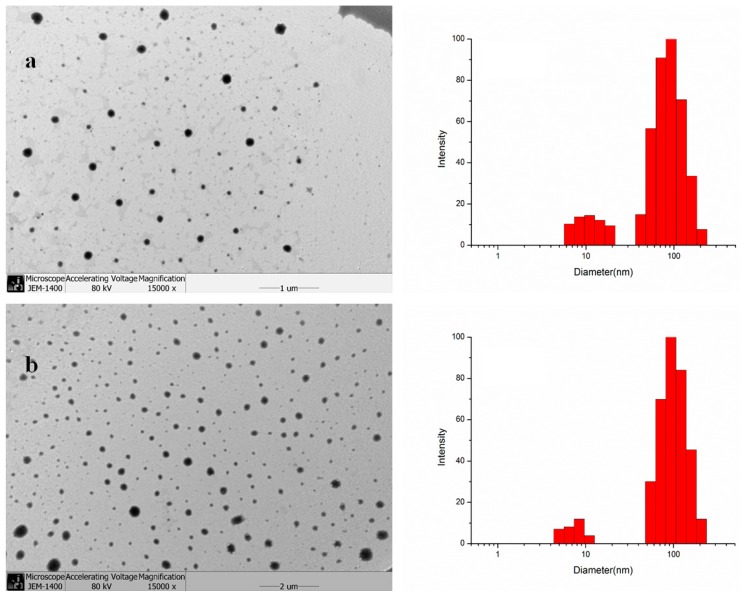
Transmission electron microscopy and size distribution of (**a**) GAP NPs and (**b**) CUR-GAP NPs.

GAP and CUR-GAP NPs were spherical in shape with a smooth surface (Figure 3), so GAP and CUR-GAP NPs could form self-aggregated NPs in aqueous media, and the size could be controlled by the DS (Table 1). The DLS results show a small population of GAP or CUR-GAP micelles around 10 nm, in size, which may be particles of GA and pullulan alone. Of course, fewer particles with higher DS or lower LC% were relatively small.

As shown in Table 1, the diameter decreased but LC% increased with increasing DS. This phenomenon can be explained by the fact that more hydrophobic groups are beneficial to form more compact hydrophobic cores and load more hydrophobic drugs [38,39,40], resulting in small size and high drug loading capacity. When the ratio of CUR to GAP weight was increased from 5%–20%, CUR loading capacity and diameter of CUR-GAP NPs increased from 4.97%–10.29% and 97.1 nm–123.6 nm respectively, while encapsulation efficiency decreased from 78.3%–68.9%. Therefore, considering the size and the drug-loading properties, we chose a weight ratio of 10% to prepare CUR-GAP NPs.

### 2.3. In Vitro Drug Release

**Figure 4:**
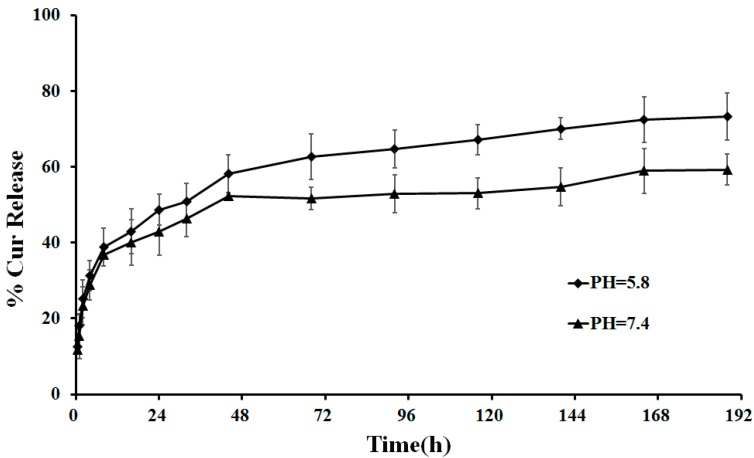
*In vitro* release of CUR-GAP NPs under acidic (pH 5.8) and neutral (pH 7.4) conditions at 37 °C. Data are mean ± SD, n = 3.

We chose a simulated physiological environment (pH 7.4) and acidic tumor extracellular pH (pH 5.8) to study CUR release at 37 °C for 8 days. We found a burst release within the first 8 h both at pH 7.4 and 5.8 (Figure 4), which might be due to the surface-absorbed CUR present in the NPs. Thereafter, CUR release was slow and uniform, which could be due to the diffusion of the entrapped drug in NPs. Thus, GAP NPs have potential as a sustained release carrier for CUR. The amount of CUR released was greater in acidic media than in the physiological environment, being approximately 73% at pH 5.8 and 59% at pH 7.4, so the CUR release from CUR-NPs is pH-dependent, which can be due to the partial acidic hydrolysis of the ester group, as reported in the literature [41]. Therefore, the release of CUR under neutral conditions such as into blood plasma and normal liver tissue would be minimized, thus reducing the systemic distribution of CUR during drug delivery.

### 2.4. Solubility and Stability of CUR

**Figure 5:**
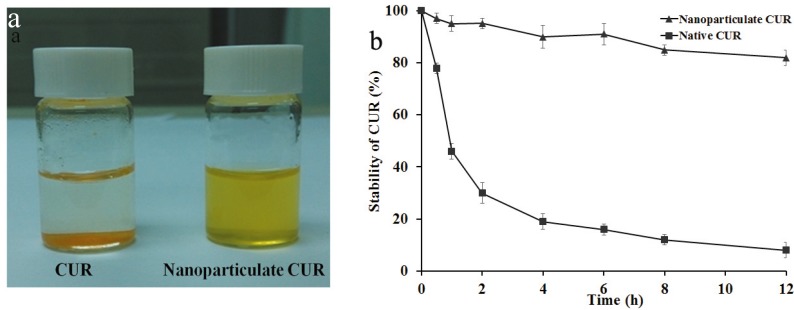
Solubility (**a**) and stability (**b**) of CUR and CUR NPs in phosphate buffered saline at 37 °C. Data are mean ± SD, n = 3.

CUR has potential therapeutic value for cancer, neoplastic, neurological, cardiovascular, pulmonary and metabolic diseases [42,43], but the low solubility in water and instability and biodegradation at physiological pH restrict its clinical applications [7]. CUR-GAP NPs showed a clear, well-dispersed formulation with the natural color of CUR, whereas native CUR was poorly soluble in water, with CUR powder precipitated on the bottom of flasks (Figure 5). CUR-GAP NPs had no significant degradation after 12 h incubation at pH 7.4. However, native CUR underwent rapid degradation in the first 2 h, and less than 10% CUR was left after the same incubation. CUR NPs could increase the aqueous solubility and stability of CUR in physiological environment, which is significant for promoting further use of CUR in medical fields.

### 2.5. Cell Uptake of CUR-GAP NPs

**Figure 6:**
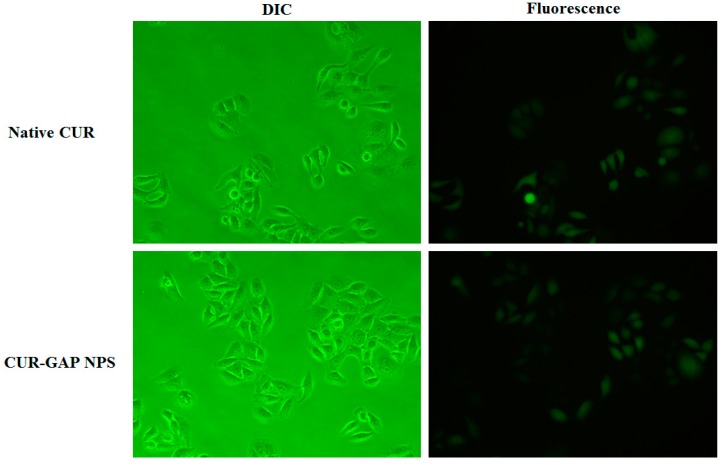
Differential interference contrast and fluorescence images of internalization of native CUR and CUR NPs by HepG2 cells.

By taking advantage of the photochemical properties of CUR, we studied the intracellular uptake by fluorescence microscopy. The fluorescence signals from HepG2 cells treated with CUR and CUR-GAP NPs were almost equal (Figure 6). However, nanoparticulate CUR can be directly dissolved in water with higher stability. This is significant for promoting further applications of CUR.

CUR with a molecular weight of about 368 can enter the cell via passive diffusion [44], while CUR-GAP NPs were less likely to enter cell by passive diffusion because of the large molecular weight. In the cell uptake study, HepG2 cells were incubated with 20 μM CUR and nanoparticulate CUR for only 2 h. According to the CUR release at pH 7.4, only a small amount of CUR (less than 20%) was released during the first 2 h, which is not sufficient enough for the released CUR to achieve the same level of fluorescence signals. Therefore, CUR-GAP NPs may be internalized by HepG2 cells by endocytosis and then degraded to release encapsulated CUR.

### 2.6. In Vitro Cytotoxicity Assay

**Figure 7:**
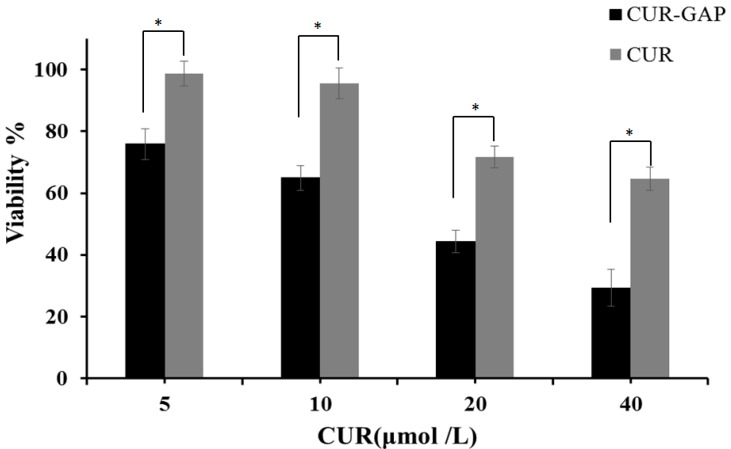
*In vitro* viability of CUR and CUR-GAP NPs in HepG2 cells at 24 h. Data are mean ± SD (n = 6), * *p* < 0.01.

The cytotoxicity of NPs was evaluated by MTT cell viability assay in HepG2 cells, human liver cancer cells with GA receptors [45]. HepG2 cells were incubated with the CUR-loaded micelles at a dose equivalent to that of the free CUR. Blank GAP micelles showed no significant cytotoxicity at the highest concentration (1 mg/mL) in HepG2 cells after 24 h incubation (data not shown) and the cytotoxicity of CUR-GAP NPs and free CUR increased with increasing CUR concentration (Figure 7), so cytotoxicity was dominated by CUR and not blank NPs. Growth of HepG2 cells was inhibited more with CUR-GAP micelles than free CUR. CUR-GAP NPs may have high affinity to hepatocytes because of the abundant GA receptors on hepatocyte membranes [30] and the inherent affinity of pullulan for the liver. Therefore, GAP NPs may not only be a safety drug carrier, but also improve the drug efficacy because of its target and NP characteristics.

## 3. Experimental

### 3.1. Materials

Pullulan (Mw = 200,000) was from Hayashibara (Tokyo, Japan). 4-Dimethylaminopyridine (DMAP), *N*,*N*'-carbonyldiimidazole (CDI) and diphenyltetrazolium bromide (MTT) were from Sigma Co. (St. Louis, MO, USA). GA, CUR and dimethylsulfoxide, anhydrous were from Aladdin Reagent Co. (Shanghai, China). All other chemicals were of analytical grade.

### 3.2. Synthesis and Characterization of GA-Modified Pullullan (GAP)

GA can be directly attached to pullulan by an esterification reaction. GAP was synthesized as follows: pullulan was dissolved in dry DMSO, then different amounts of GA were added. DMAP and CDI were added at a molar ratio of DMAP to CDI to GA of 1:1:1. After stirring at 50 °C for 48 h, the solution was precipitated in ethanol and the precipitate was washed with ethanol, tetrahydrofuran and diethyl ether separately. Finally, the precipitate was redissolved in water and lyophilized to obtain the white, cotton-wool-like product of GAP. The chemical structure of GAP and DS, defined as the number of GA groups per 100 glucose units pullulan, was analyzed by FT-IR (KBr pellets, Nicolet iS5) and ^1^H-NMR (DMSO-d_6_, Agilent DD2, 600 MHz).

### 3.3. Preparation of GAP Self-aggregated NPs

The self-assembled NPs were prepared by the dialysis method [46]. Briefly, GAP was suspended in DMSO under gentle shaking at 37 °C until it was completely dissolved and then dialyzed against 2000 mL of distilled water for 3 days with 10 exchanges by using a dialysis bag (molecular weight cut-off 8000–14,000,Union Carbide Corporation, Chicago, IL, USA) to remove DMSO. Then, the solution was sonicated 3 times by use of a probe type sonifier (Automatic Ultrasonic Processor UH-500A, Shanghai, China) at 100 W for 2 min each in an ice water bath and with pulsing (pulse on 2.0 s, off 2.0 s) to protect against heat build-up during sonication. The self-assembled NPs were then passed through a membrane filter (pore size: 0.45 µm, Millipore, Billerica, MA, USA) and stored at 4 °C.

### 3.4. Transmission Electron Microscopy (TEM) and Dynamic Light Scattering (DLS)

To observe the morphologic features of GAP and CUR-GAP NPs, one drop of the NP suspension was placed on carbon-coated 300 mesh grids. Then, the grids were air-dried, stained with 2% phosphotungstic acid solution for 2 min and examined by TEM (JEM-1400, Tokyo, Japan) at an accelerating voltage of 80 KV. The size of CUR-GAP and GAP NPs was investigated by DLS (BI-90US, Tokyo, Japan). The NP suspensions were filtered with a 0.45 μm filter, and each batch was analyzed in triplicate.

### 3.5. Preparation and Characterization of CUR-Loaded GAP NPs

GAP solution (1 mg/mL) was prepared as described and stirred in an ice bath for 24 h, then different amounts of CUR in DMSO were added. The solutions were dialyzed against 2 L distilled water for 3 days with 10 exchanges by use of a dialysis bag, sonicated by use of a probe type sonicator at 100 W for 2 min to obtain CUR-loaded GAP self-aggregated NPs and centrifuged at 3000 rpm for 10 min to remove the unloaded CUR and large NPs. The supernatant was lyophilized and stored at 4 °C.

Lyophilized CUR-GAP NPs (2–3 mg) were dissolved in 10 mL methanol, then gently shaken on a shaker for 12 h at room temperature to completely leach out CUR from CUR-GAP NPs in methanol. Solutions were centrifuged at 14,000 rpm and supernatant was determined by use of a microplate reader (SpectraMax M2, Molecular Devices, Sunnyvale, CA, USA) at 425 nm. Empty GAP NPs were used as the blank test. The CUR loading capacity (LC) and encapsulation efficiency (EE) were calculated as follows:

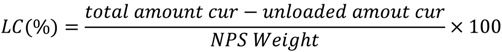
(2)

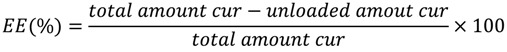
(3)

The morphology and particle size of CUR-GAP NPs were studied by the above methods.

### 3.6. Solubility and Stability of CUR

Native CUR and nanoparticulate CUR of an equivalent quantity of CUR were dissolved in water to compare aqueous solubility. Native CUR and nanoparticulate CUR at a fixed CUR concentration of 15 µg/mL were prepared in phosphate buffered saline (PBS: 0.01 M, pH 7.4) with less than 5% by volume of methanol to improve the solubility of native CUR in PBS (0.01 M, pH 7.4), and shaken at 200 rpm, 37 °C for 12 h [47,48]. At designated times, 900 µL methanol was added to 100 µL sample, to quantify the stability of CUR with time in PBS by a microplate reader at 425 nm.

### 3.7. Release Kinetics of CUR from CUR-GAP NPs in Vitro

The *in vitro* release of CUR from CUR-GAP NPs was performed in PBS at simulated physiological environment (pH 7.4) and acidic tumor extracellular pH (pH 5.8). CUR-GAP NPs of 100 mg were dissolved in 15 mL PBS and the solution was divided into 30 Eppendorf tubes (0.5 mL each) [12,47,48]. The samples were shaken at 200 rpm at 37 °C. Free CUR is completely insoluble in water; therefore, at designated times, the solution was centrifuged at 3000 rpm for 10 min to separate the released CUR from the CUR-GAP NPs. The released CUR was re-dissolved and diluted in methanol and determined by a microplate reader at 425 nm. All procedures were carried out in triplicate.

### 3.8. In Vitro Cellular Uptake

HepG2 cells were seeded in 24-well plates at 1 × 10^4^ cells per well in 1 mL growth medium to study intracellular CUR fluorescence. After 24-h incubation, the attached cells were incubated with 20 μM native CUR and CUR-GAP NPs for 2 h, then washed 3 times with PBS (0.01 M, pH 7.4) to remove excess NPs or CUR and 1 mL PBS was added for fluorescence microscopy studies. The free CUR was dissolved in less than a thousandth by volume of DMSO to improve the solubility of native CUR.

### 3.9. In Vitro Cytotoxicity

The cytotoxicity of GAP NPs and CUR-GAP NPs and free CUR was determined by measuring the inhibition of cell growth by tetrazolium dye (MTT) assay as described [49]. Briefly, human hepatocellular carcinoma (HepG2) cells were seeded at 1 × 10^3^ cells/well in 96-well plates. After 24 h incubation, cells were treated with serial dilutions of GAP NPs, CUR-GAP NPs and free CUR in serum-free medium for 24 h, then with MTT solution (20 µL, 5 mg/mL in PBS) for 4 h at 37 °C. The resulting formazan was dissolved in DMSO (150 µL) and measured at 490 nm by use of a microplate reader. The cellular growth inhibition was calculated by the following equation:

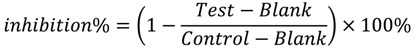
(4)

We used untreated cells as the control (100% survival) and non-cell wells as the blank to substrate solvent absorbance. All experiments were replicated six times.

## 4. Conclusions

In this study, we describe a simple method to synthesize and characterize a novel GAP NP carrier of encapsulated CUR that could improve the solubility and stability of CUR in a simulated physiological environment. The CUR-GAP NPs showed a sustained and pH-dependent release behavior, which is key to reducing the systemic distribution of CUR by reducing the release of CUR under neutral conditions such as into blood plasma and normal liver. HepG2 cells treated with both free and encapsulated CUR showed green fluorescence, which confirmed the successful delivery of CUR into cells. Furthermore, the CUR-GAP NPs could significantly improve the water solubility, stability and cytotoxicity of CUR in HepG2 cells *in vitro*, which may due to the liver target of GA and the inherent affinity for the liver from pullulan.
